# Daikenchuto, a traditional Japanese herbal medicine, for the maintenance of surgically induced remission in patients with Crohn’s disease: a retrospective analysis of 258 patients

**DOI:** 10.1007/s00595-013-0747-6

**Published:** 2013-10-17

**Authors:** Amane Kanazawa, Minako Sako, Masakazu Takazoe, Tokuma Tadami, Takaaki Kawaguchi, Naoki Yoshimura, Kinya Okamoto, Tetsuo Yamana, Rikisaburo Sahara

**Affiliations:** 1 Department of Coloproctology, Social Health Insurance Medical Center, 3-22-1 Hyakunincho, Shinjuku-ku, Tokyo 169-0073 Japan; 2 Inflammatory Bowel Disease Center, Social Health Insurance Medical Center, 3-22-1 Hyakunincho, Shinjuku-ku, Tokyo 169-0073 Japan

**Keywords:** Crohn’s disease, Daikenchuto, Reoperation rate, 5-aminosalicylic acid

## Abstract

**Purpose:**

Despite numerous studies, the best postoperative therapy for Crohn’s disease is still undefined. We retrospectively evaluated the effects of postoperative maintenance therapy with daikenchuto, a traditional Japanese Kampo medicine, on the reoperation rate at 3 years in patients with Crohn’s disease.

**Methods:**

A total of 258 patients who underwent surgery for Crohn’s disease were identified for the study. For the prevention of postoperative recurrence, patients were stratified to receive 5-aminosalicylic acid, azathioprine or daikenchuto, and their effects on preventing reoperation at 3 years were evaluated.

**Results:**

Of the 258 patients, 44 required reoperation with intestinal resection within 3 years due to disease recurrence. The 3-year reoperation rate was significantly lower in the postoperative daikenchuto group than in the non-daikenchuto group (11.3 vs. 24.5 %, *P* = 0.01), and was similarly significantly lower in the postoperative 5-aminosalicylic acid group than in the non-5-aminosalicylic acid group (14.8 vs. 29.6 %, *P* = 0.0049). A multivariate Cox analysis showed that postoperative daikenchuto (*P* = 0.035) and postoperative 5-aminosalicylic acid (*P* = 0.022) were significantly and independently associated with the rate of reoperation at 3 years in patients with Crohn’s disease.

**Conclusion:**

We propose that continuous daikenchuto therapy is a clinically useful and feasible maintenance therapy for the prevention of postoperative reoperation in patients with Crohn’s disease.

## Introduction

Crohn’s disease (CD) is a chronic transmural inflammatory disease that frequently relapses at the site of anastomosis, making prevention of recurrence a challenging clinical problem. Postoperative recurrence in the absence of treatment has been reported to occur in approximately 65–90 % of patients within 12 months and in 80–100 % within 3 years after the initial surgery [1]. Nearly 80 % of all patients with Crohn’s disease undergo intestinal resection during their lifetime [2], and 11–32 % require reoperation within 5 years [3]. This high rate of reoperation severely compromises the quality of life of patients with CD, and has prompted the development of many drug therapies for the prevention of disease recurrence and reoperation [4]. For instance, biological drugs such as infliximab form the mainstay of treatment for patients with moderate to severe CD, while 5-aminosalicylates (5-ASA) and steroids with anti-inflammatory or immunomodulatory effects are given to those with mild to moderate disease for long-term maintenance of remission [5, 6]. Despite numerous studies and advances in the medical treatment of CD, the best postoperative prophylactic therapy is still undefined. In Japan, enteral nutrition and traditional Japanese Kampo medicines, such as daikenchuto (DKT), are often used as invaluable adjuncts to drug therapy; however, there have been no objective reports on their clinical efficacy.

DKT has been traditionally used for the treatment of postoperative ileus, and its efficacy for this condition has been documented in several clinical trials [7]. Yoshikawa et al. [8] showed that DKT significantly suppressed the CRP level and shortened the time until first flatus in colorectal cancer patients following laparoscopic colectomy. One of the best pieces of evidence supporting its postoperative use comes from a randomized, double-blind, placebo-controlled trial that revealed an acceleration of intestinal motility on scintigraphy in healthy volunteers [9]. In animal CD models, DKT has been shown to either directly or indirectly reduce inflammation and restore the blood flow to ischemic segments of the colon by promoting endogenous adrenomedullin (ADM) release, thereby contributing to the prevention of disease recurrence [10–12]. Chikakiyo et al. [13] reported that DKT suppressed CPT-11-induced inflammatory cytokines and apoptosis.

Although the reoperation rate at 5 years is typically assessed to determine the outcome of postoperative maintenance therapy, i.e., for monitoring postoperative recurrence, we used the 3-year reoperation rate to obtain an early appraisal of the efficacy of these therapies, and also because the 3-year reoperation rate has been previously reported to be a good predictor of the 5-year reoperation rate [14]. In addition, we surmised that since most clinical relapses after curative surgery occur within the first 3 years [15], the absence of reoperation may be a relevant marker of efficacy. Thus, the purpose of this study was to retrospectively determine the efficacy of DKT compared with other postoperative maintenance therapies by evaluating the reoperation rates at 3 years in patients with CD.

## Patients and methods

### Patients

A total of 258 patients who underwent surgery for CD between January 2006 and December 2007 in the Department of Coloproctology Center, Social Health Insurance Medical Center, were identified for the study. All clinical data were obtained from medical records and the prospectively maintained CD patient database and were retrospectively analyzed. The study was approved by the Ethics Committee of the Social Health Insurance Medical Center. The characteristics of the patients who received active postoperative therapy are summarized in Table 1.

**Table 1:** Characteristics of patients who received active postoperative therapy

Variables/categories	No. of patients (%)
Age at operation (mean ± SD)	33.7 ± 8.5
Duration of disease (mean ± SD)	11.9 ± 7.2
Sex	
Female	70 (27.1)
Male	188 (72.9)
Previous operations	
Yes	120 (46.5)
No	138 (53.5)
Disease location	
Small bowel	83 (32.2)
Other^a^	175 (67.8)
Disease behavior	
Penetrating	161 (62.4)
Other	97 (37.6)
Preoperative IOIBD assessment score	
<2	76 (29.5)
≥2	182 (70.5)
Preoperative albumin (g/dl)	
≤4.0	221 (85.7)
>4.0	37 (14.3)
Postoperative residual lesion	
Yes	173 (67.1)
No	85 (32.9)
Postoperative 5-ASA	
Yes	175 (67.8)
No	83 (32.2)
Postoperative AZA	
Yes	35 (13.6)
No	223 (86.4)
Postoperative DKT	
Yes	100 (38.8)
No	158 (61.2)
Reoperation within 3 years	
Yes	44 (17.1)
No	214 (82.9)

*SD* standard deviation, *IOIBD assessment score* International Organization for the Study of Inflammatory Bowel Disease assessment score, *5*-*ASA* 5-aminosalicylic acid, *AZA* azathioprine, *DKT* daikenchuto

^a^Small and large bowel + large bowel only

### Study design

Surgical resection was indicated for patients based on their preoperative clinical course and radiological, endoscopic and CT results. In our study, previous operations as a risk variable pertained only to abdominal surgeries performed for the treatment of diseases or complications due to CD. For this reason, surgical treatments for perianal disease were not accounted for as previous operations. Similarly, appendectomy was not regarded as previous surgery, because the procedure was performed in patients who did not have CD at the time of appendectomy, but developed the condition several years later. The disease location was defined as either confined to the small bowel or affecting both the small and large bowels. The disease behavior at the time of surgery was defined as either penetrating or other types (including non-penetrating, stricturing or non-stricturing). The International Organization for the Study of Inflammatory Bowel Disease (IOIBD) assessment score was recorded within 3 months prior to surgery and graded as <2 or ≥2. The preoperative serum albumin level was defined as either low (≤4.0 g/dl) or normal (>4.0 g/dl).

At our institution, the general recommendations for postoperative medical treatment are as follows: 5-ASA as first-line therapy for the maintenance of surgically induced remission, azathioprine (AZA) for patients with postoperative residual lesions or a history of repeat surgeries within a short period of time and DKT for patients with severe adhesion at the time of laparotomy or a history of previous surgery. In clinical practice, the internist selected the appropriate therapy according to these recommendations, while taking into account each patient’s specific condition. Reoperation has been typically reserved for patients who are refractory to these therapies and on the basis of careful evaluation of the clinical, endoscopic and radiological findings.

For the prevention of postoperative recurrence, patients were stratified to receive 5-ASA (2250–3000 mg/day), AZA (50–100 mg/day) or DKT (7.5–15 g/day). These therapies were initiated within 1 month after surgery and continued for at least a year. The detailed characteristics of the patients who received active postoperative therapy appear in Table 1. The cumulative reoperation rates were calculated for the DKT group (100 patients), non-DKT group (158 patients), 5-ASA group (175 patients) and non-5-ASA group (83 patients).

### Daikenchuto

**Fig. 1:**
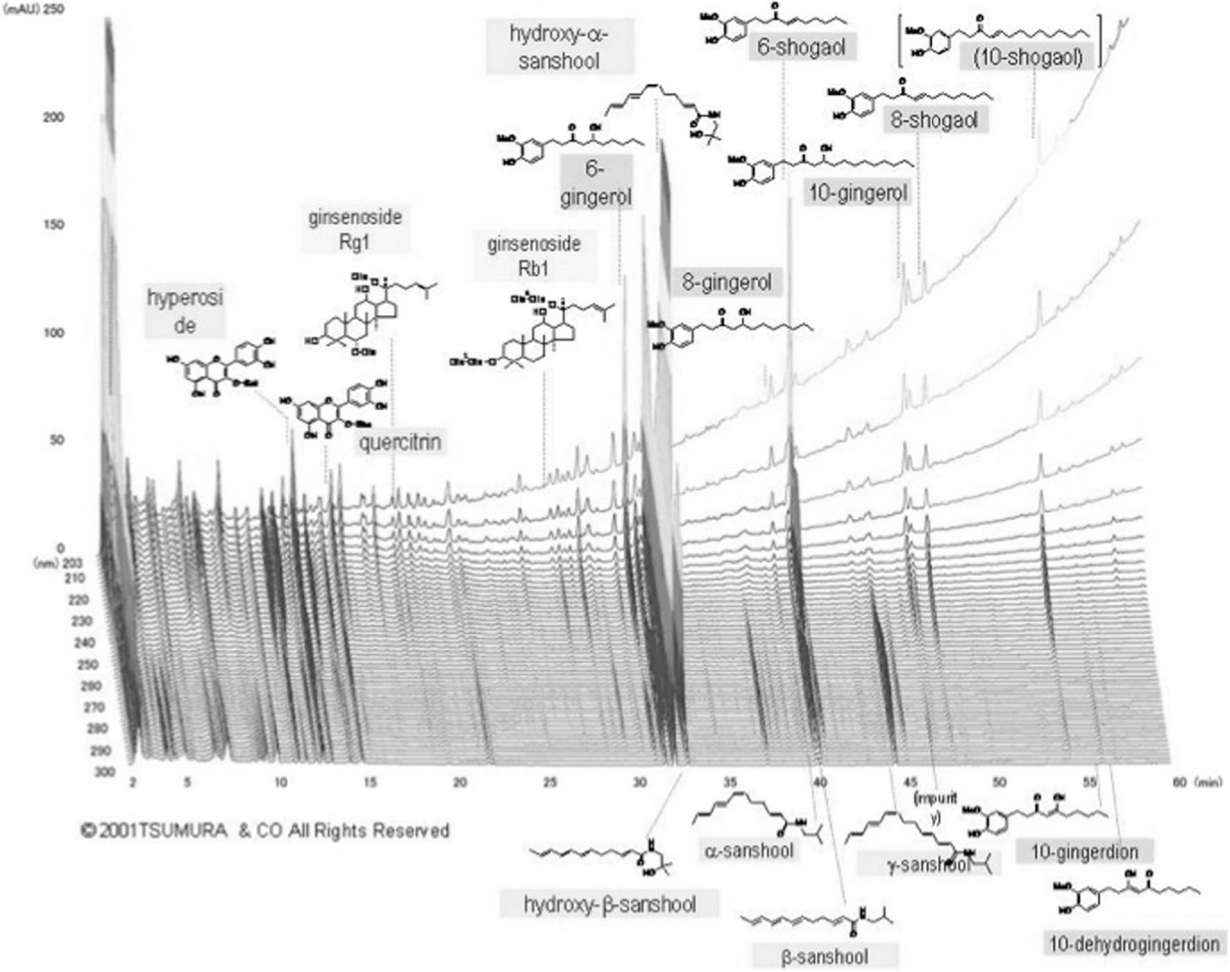
The 3-D high-performance liquid chromatography (HPLC) profile of daikenchuto

DKT is composed of three crude drugs in fixed proportions: Zingiberis Rhizoma (processed ginger, 5.0 g), Ginseng Radix (Panax ginseng, 3.0 g) and Zanthoxyli Fructus (Japanese pepper, 2.0 g). The dried extract powder of DKT was supplied by Tsumura & Co. (Tokyo, Japan). The DKT extract powder was prepared by decocting the three crude drugs in a tenfold volume of purified water at 95 °C for 1 h, which was then filtered and spray-dried to yield the extract powder. At the end, maltose syrup powder was added at a ratio of 1:8. For the analysis of DKT components, 1.0 g of the extract powder was extracted with 20 ml of methanol under ultrasonication for 30 min and then centrifuged at 3000 rpm for 5 min. The supernatant was filtered through a 0.45-μm membrane, and 30 μL of the filtrate was subjected to a high-performance liquid chromatography (HPLC) analysis. The three-dimensional HPLC profile of DKT is shown in Fig. 1.

### Statistical analysis

The descriptive data were reported as the means, standard deviations, ranges or numbers of patients and percentages. Categorical variables were compared by the *χ* ^2^ test. The 3-year cumulative reoperation rate was estimated by the Kaplan–Meier method and compared between groups using the log-rank test. The univariate and multivariate analyses were performed by using a Cox proportional hazard ratio model to identify the independent predictors of reoperation. The risk variables considered were the age, sex, previous operations, disease location, disease behavior, preoperative IOIBD score, preoperative serum albumin level, postoperative residual lesions and postoperative maintenance therapy (postoperative 5-ASA, postoperative DKT or postoperative AZA). Differences were considered to be significant for values of *P* < 0.05. Each statistical analysis was performed using the Dr. SPSS II program, version 11.0.1 J for Windows (SPSS INC., Chicago, IL).

## Results

### Characteristics of patients

Table 1 describes the patient characteristics and clinical predictors of reoperation. The mean age at first surgery was 33.7 years (range, 16–65) and the mean duration of disease from onset to initial surgery was 11.9 years (range, 0–37). A disproportionate number of male patients compared to female patients had CD (188 vs. 70) and over half of all patients had undergone a previous abdominal surgery. A total of 336 surgical resections were performed, which included partial small bowel resection (110), ileocecal resection (53), ileostomy and colostomy (47), hemicolectomy (28), partial colectomy (28), total colectomy (26), strictureplasty (14), ileocolectomy (12), Miles surgery (6), anterior resection (5) and other procedures (7).

### Three-year reoperation rates

**Fig. 2:**
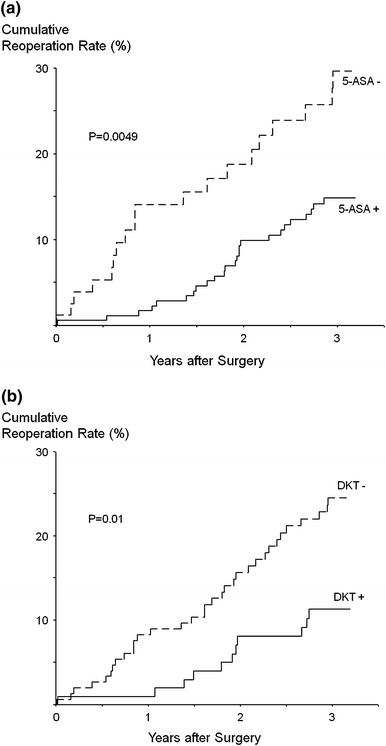
The postoperative reoperation rates between the 5-aminosalicylic acid (5-ASA) group (*n* = 175) and the non-5-ASA group (*n* = 83) (**a**), and between the daikenchuto (DKT) group (*n* = 100) and the non-DKT group (*n* = 158) (**b**). Significant differences were found in the 3-year postoperative reoperation rates between the 5-ASA group and non-5-ASA group (14.8 vs. 29.6 %, *P* = 0.0049), and between the DKT group and non-DKT group (11.3 vs. 24.5 %, *P* = 0.01)

Of the 258 patients who underwent surgery, 44 (17.1 %) required reoperation with intestinal resection within 3 years due to disease recurrence. The cumulative reoperation rate was calculated for the DKT group (100 patients) vs. the non-DKT group (158 patients), and for the 5-ASA group (175 patients) vs. the non-5-ASA group (83 patients). The 3-year reoperation rate was significantly lower in the postoperative 5-ASA group than in the non-5-ASA group (14.8 vs. 29.6 %, *P* = 0.0049 by the log-rank test, Fig. 2a). Similarly, a significantly lower reoperation rate was observed in the postoperative DKT group than in the non-DKT group (11.3 vs. 24.5 %, *P* = 0.01, Fig. 2b).

The Cox’s univariate regression analysis showed that the 3-year postoperative reoperation rate correlated significantly with postoperative 5-ASA and postoperative DKT administration (Table 2). Further, the Cox’s multivariate analysis showed that postoperative 5-ASA (*P* = 0.022) and postoperative DKT (*P* = 0.035) were significant and independent predictors related to reoperation at 3 years in patients with CD (Table 3). Statistical significance was not found for other variables such as age, sex, surgery history, disease location, disease behavior, preoperative IOIBD score, preoperative serum albumin level and postoperative AZA treatment.

**Table 2:** The results of the univariate analysis of variables predicting reoperation within 3 years of resection

Variables/categories	*n*	Hazard ratio	95 % CI	*P* value
Age (years)				
<40	55	1		
≥40	203	1.653	0.768–3.556	0.199
Sex				
Female	70	1		
Male	188	1.254	0.583–2.697	0.563
Previous operations				
Yes	120	1		
No	138	1.342	0.735–2.447	0.338
Disease location				
Small bowel	83	1		
Other^a^	175	1.465	0.74–2.899	0.273
Disease behavior				
Penetrating	161	1		
Other	97	1.233	0.676–2.25	0.494
Preoperative IOIBD assessment score				
<2	76	1		
≥2	182	1.651	0.794–3.435	0.18
Preoperative serum albumin (g/dl)				
≤4.0	221	1		
>4.0	37	1.052	0.469–2.361	0.901
Postoperative residual lesion				
Yes	173	1		
No	85	1.583	0.872–2.875	0.131
Postoperative 5-ASA				
Yes	175	1		
No	83	2.302	1.266–4.185	0.006
Postoperative AZA				
Yes	35	1		
No	223	3.892	0.942–16.078	0.06
Postoperative DKT				
Yes	100	1		
No	158	2.379	1.202–4.709	0.013

*IOIBD assessment score* International Organization for the Study of Inflammatory Bowel Disease assessment score, *5*-*ASA* 5-aminosalicylic acid, *AZA* azathioprine; *DKT* daikenchuto, *CI* confidence interval

^a^Small and large bowel + large bowel only

**Table 3:** The results of the multivariate analysis of variables predicting reoperation within 3 years of resection

Variables/categories	Hazard ratio	95 % CI	*P* value
Postoperative 5-ASA (Yes/No)	2.027	1.105–3.717	0.022
Postoperative DKT (Yes/No)	2.111	1.056–4.222	0.035

*5*-*ASA* 5-aminosalicylic acid, *DKT* daikenchuto, *CI* confidence interval

### Adverse events

The known adverse effects of DKT include interstitial pneumonia, hepatic dysfunction, rashes, hives and gastrointestinal distress such as stomach discomfort, nausea and vomiting. With the exception of a minor side effect such as diarrhea, no serious adverse events were observed during the study.

## Discussion

To date, a multitude of clinical predictive factors for postoperative recurrence in CD have been postulated, including the age at disease onset, duration of disease before resection, sex, family history of CD, smoking habit, disease location, genetic factors, prophylactic treatment, involvement of disease at the resection margin, penetrating disease behavior, length of the resected bowel, anastomotic technique, histological findings, blood transfusion and postoperative complications [2, 3, 16, 17], although the results from various studies have been largely heterogeneous. Our results showed that clinical predictors such as the age, sex, previous operations, disease location, disease behavior, preoperative IOIBD score, preoperative serum albumin level, postoperative residual lesions and postoperative AZA therapy had no significant effect on surgical recurrence at 3 years, except for postoperative maintenance therapy with 5-ASA or DKT. In this study, 258 patients who underwent surgical resection were retrospectively analyzed for the reoperation rate at 3 years after stratification by postoperative therapy: these were the DKT, non-DKT, 5-ASA or non-5-ASA groups. We found that the 3-year reoperation rate was significantly lower in the DKT group than in the non-DKT group (*P* = 0.01); a significantly lower reoperation rate was also observed in the 5-ASA group compared to the non-5-ASA group (*P* = 0.0049). Interestingly, our results indicated a near equivalent reoperation rate between the DKT and 5-ASA groups (11.3 vs. 14.3 %), thus supporting the potential use of DKT for remission maintenance in patients with CD. Because most relapses after curative surgery occur within the first 3 years [15], we consider our results in this respect to be cogent.

Effective postoperative drug therapy is critical in CD management, as the endoscopic, clinical and surgical recurrence rates are high. Several studies have evaluated the efficacy of various pharmacotherapies for the prevention of postoperative recurrence in patients with CD, including antibiotics, metronidazole, 5-ASA, AZA, enteral nutrition and 6-mercatopurine [5, 6]. A meta-analysis of five randomized controlled trials of 729 patients showed that mesalazine treatment significantly reduced the risk of clinical recurrence at 1 year after surgery [18]. Our current findings corroborate the efficacy of 5-ASA products, which have been used as the mainstay of remission induction therapy for mild to moderate disease, in significantly reducing the reoperation rate at 3 years. The purported mechanisms by which 5-ASA drugs confer therapeutic benefit include direct scavenging of free radicals [19, 20] and inhibition of leukotriene production [21] and inflammatory processes [22, 23].

In Japan, DKT is used with the intent to prolong CD remission, but data on its beneficial effect on prolonging remission are scarce. There are, however, many reports on the effect of DKT for other gastrointestinal disorders [9, 10, 24, 25]. For instance, Itoh et al. [7] have shown that oral administration of DKT is clinically effective for postoperative ileus and reducing the recurrence and the need for further surgery. A more recent study has shown that DKT has a clinically significant pro-motility effect on the small bowel and ascending colon transit in healthy subjects in a randomized, parallel-group, double-blind, placebo-controlled, dose–response study [9]. In contrast to 5-ASA agents, DKT appears to work by upregulating endogenous epithelial ADM, which has been shown in mouse and rat models of CD to attenuate mucosal damage and inflammatory adhesions, suppress mucosal proinflammatory cytokine (TNF-α and IFN-γ) release and improve the blood flow in ischemic segments [15, 26]. More recent data from the study by Kono et al. [27] identified the principal component in Japanese pepper, hydroxyl-α-sanshool, as the key constituent mediating endogenous ADM release. Given that microvascular dysfunction of the CD intestine, particularly at the anastomotic site and along the mesenteric margin, is likely involved in the recurrence of CD [28], the anti-inflammatory and vasodilatatory effects of DKT within the colonic microvasculature via the enhanced ADM release predict a favorable clinical outcome [10–12]. Thus, the clinical usefulness of DKT, whose mechanistic actions differ from those of 5-ASA products, may herald the expansion of new therapeutic strategies to optimize the postoperative outcomes.

Although DKT has been reported to produce adverse effects such as interstitial pneumonia, hepatic dysfunction, rashes, hives and GI distress, such as stomach discomfort, nausea and vomiting [10, 26], no serious DKT-related adverse events were observed during the study period. Our analysis also suggests that DKT may be among the limited remission induction therapies capable of being continuously administered.

In summary, our retrospective analysis of 258 patients with CD showed that the efficacy of DKT rivals that of 5-ASA for preventing reoperation in patients with mild to moderate disease, indicating that DKT may be a viable option for the postoperative management of CD. In addition, our current data may lend further credence to the safety and cost-effectiveness of DKT as a maintenance therapy for CD. Further prospective, randomized, placebo-controlled trials are needed to confirm our findings in this subset of patients to define its value in the postoperative period.

## Conclusion

This report summarizes the first retrospective analysis of the efficacy and safety of long-term DKT administration for preventing reoperation in patients with CD. On the basis of our findings that DKT significantly reduces the 3-year reoperation rate as robustly as 5-ASA, we propose that continuous DKT administration is a clinically useful and feasible form of maintenance therapy for patients with mild to moderate CD.
